# The Society for Immunotherapy of Cancer consensus statement on immunotherapy for the treatment of prostate carcinoma

**DOI:** 10.1186/s40425-016-0198-x

**Published:** 2016-12-20

**Authors:** Douglas G. McNeel, Neil H. Bander, Tomasz M. Beer, Charles G. Drake, Lawrence Fong, Stacey Harrelson, Philip W. Kantoff, Ravi A. Madan, William K. Oh, David J. Peace, Daniel P. Petrylak, Hank Porterfield, Oliver Sartor, Neal D. Shore, Susan F. Slovin, Mark N. Stein, Johannes Vieweg, James L. Gulley

**Affiliations:** 1 University of Wisconsin Carbone Cancer Center, 7007 WIMR, 1111 Highland Avenue, Madison, WI 53705 USA; 2 Weill Medical College of Cornell University, Laboratory of Urological Oncology E-300, 525 East 68th Street, New York, NY 10021 USA; 3 Oregon Health and Science University Knight Cancer Institute, 3181 SW Sam Jackson Park Road, Portland, OR 97239 USA; 4 Johns Hopkins University, 1650 Orleans Street Room 410, Baltimore, MD 21287 USA; 5 University of California, San Francisco, 513 Parnassus Ave, Room HSF 301, Box 1270, San Francisco, CA 94143 USA; 6 Carolina Urologic Research Center, 823 82nd Parkway, Suite B, Myrtle Beach, SC 29572 USA; 7 Memorial Sloan Kettering Cancer Center, 1275 York Avenue, New York, NY 10021 USA; 8 National Cancer Institute, National Institutes of Health, 10 Center Drive, Bethesda, MD 20892 USA; 9 Mount Sinai School of Medicine, One Gustave L. Levy Place, Box 1079, New York, NY 10029 USA; 10 University of Illinois, 840 S Wood Street, Suite 820, Chicago, IL 60612 USA; 11 Yale Cancer Center, PO Box 208032, New Haven, CT 06520 USA; 12 Alliance for Prostate Cancer Prevention, 17660 Tamiami Trail, Suite 106, Fort Myers, FL 33908 USA; 13 Tulane University School of Medicine, 1430 Tulane Ave, SL-42, New Orleans, LA 70112 USA; 14 Rutgers Cancer Institute of New Jersey, 195 Little Albany Street, New Brunswick, NJ 08903 USA; 15 Nova Southeastern University, 3200 South University Drive, Fort Lauderdale, FL 33328 USA; 16 Genitourinary Malignancies Branch, 10 Center Drive, 13N240, Bethesda, MD 20892 USA

**Keywords:** Guidelines, Immunotherapy, Prostate Cancer, Treatment

## Abstract

Prostate cancer is the most commonly diagnosed malignancy and second leading cause of cancer death among men in the United States. In recent years, several new agents, including cancer immunotherapies, have been approved or are currently being investigated in late-stage clinical trials for the management of advanced prostate cancer. Therefore, the Society for Immunotherapy of Cancer (SITC) convened a multidisciplinary panel, including physicians, nurses, and patient advocates, to develop consensus recommendations for the clinical application of immunotherapy for prostate cancer patients. To do so, a systematic literature search was performed to identify high-impact papers from 2006 until 2014 and was further supplemented with literature provided by the panel. Results from the consensus panel voting and discussion as well as the literature review were used to rate supporting evidence and generate recommendations for the use of immunotherapy in prostate cancer patients. Sipuleucel-T, an autologous dendritic cell vaccine, is the first and currently only immunotherapeutic agent approved for the clinical management of metastatic castrate resistant prostate cancer (mCRPC). The consensus panel utilized this model to discuss immunotherapy in the treatment of prostate cancer, issues related to patient selection, monitoring of patients during and post treatment, and sequence/combination with other anti-cancer treatments. Potential immunotherapies emerging from late-stage clinical trials are also discussed. As immunotherapy evolves as a therapeutic option for the treatment of prostate cancer, these recommendations will be updated accordingly.

**Electronic supplementary material:**

The online version of this article (doi:10.1186/s40425-016-0198-x) contains supplementary material, which is available to authorized users.

## Introduction

Prostate cancer remains the most commonly diagnosed malignancy in men in the United States. Despite recent decreases in screening, it is estimated that approximately 180,890 new cases will be diagnosed in 2016, accounting for 21% of newly diagnosed cancer in men [1]. Moreover, approximately 27,540 men were estimated to have died of prostate cancer in 2015, the second leading cause of cancer death among men in the United States [2]. Early detection rates combined with an indolent disease course likely account for the high 5-year survival rates approaching 100% for newly diagnosed localized (stage I and II) or regional (stage III) disease. However, approximately one-third of early stage patients will develop recurrence, often with metastatic disease. For patients with metastatic (stage IV) disease 5-year survival rates decrease to 28% [2, 3].

**Fig. 1:**
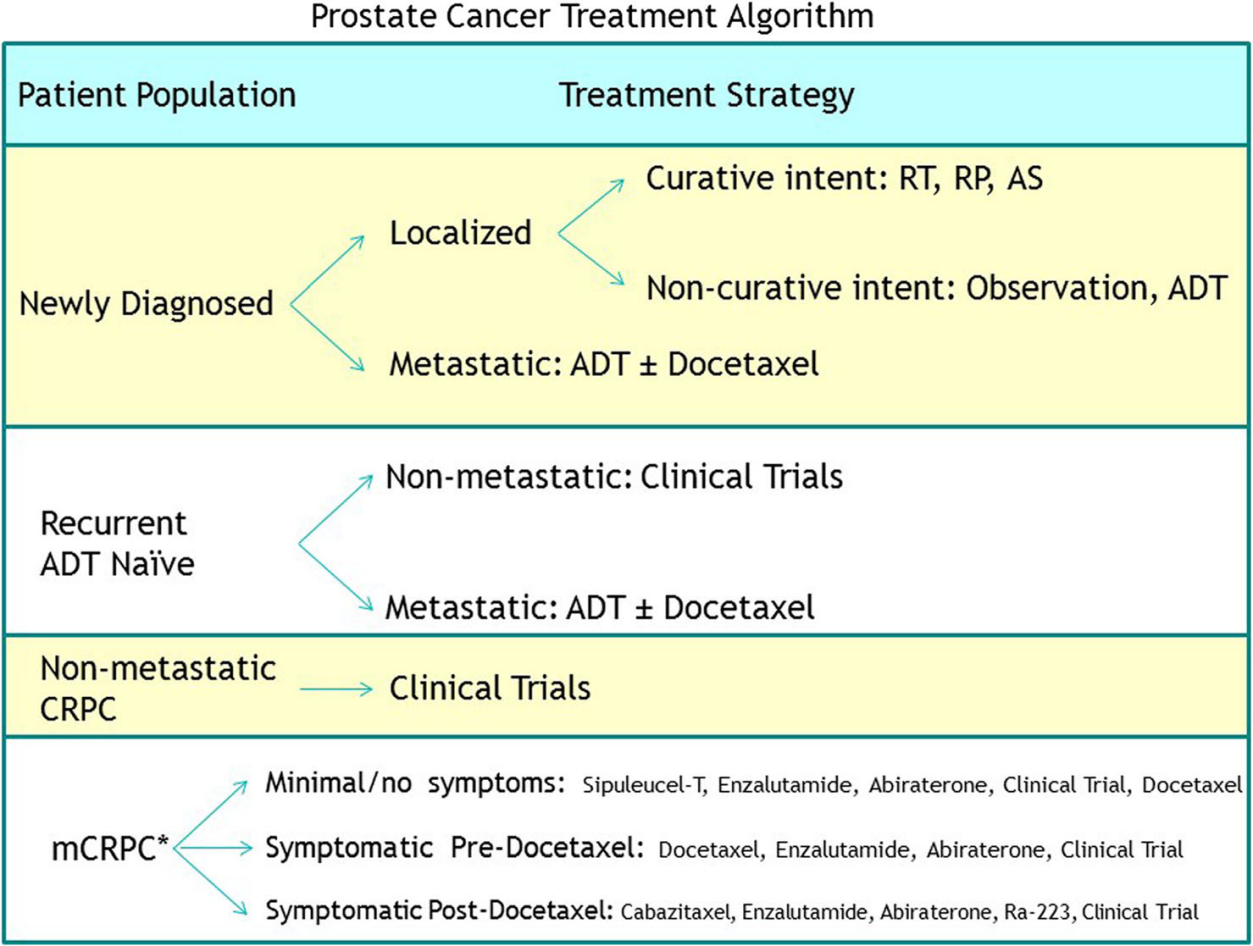
Treatment algorithm for prostate cancer. Abbreviations: radiation therapy (RT), radical prostatectomy (RP), active surveillance (AS). Asterisk (*) indicates with continuous testosterone suppression, with or without denosumab or zoledronic acid

Prostate cancer has a very heterogeneous natural history. Androgen deprivation therapy (ADT) is the mainstay of initial therapy for metastatic disease. Although prostate cancer usually initially responds to ADT, resistance eventually develops in nearly all men and the disease progresses to a state known as mCRPC. In the past 6 years, a number of therapies have been approved for mCRPC, including androgen signaling inhibitors (enzalutamide, abiraterone acetate) [4–6], cytotoxic chemotherapy (cabazitaxel) [7], a radiopharmaceutical (radium-223) [8], and immunotherapy (sipuleucel-T) [9–11]. The timing of initiation of treatment as well as the optimal sequence of these therapies has been the topic of considerable discussion and debate. Figure 1 demonstrates the current algorithm for therapy of all stages of prostate cancer. As can be noted, immunotherapy is currently employed in the setting of asymptomatic mCRPC.

There has been interest in using immunotherapy as a treatment for prostate cancer for many years. While the immunogenicity of prostate tumors was contested nearly 30 years ago, more recent evidence suggests prostate cancer is an immunologically recognized disease. T cell infiltration into prostate tumors has been identified at the time of cancer diagnosis and can be modulated by treatments such as ADT [12–14]. Cellular and humoral immune responses can be detected to prostate-specific and prostate cancer-associated proteins in patients with prostate cancer [15, 16]. Moreover, the findings of decreased MHC class I expression on advanced prostate tumors and defects in T cell signaling in patients with advanced disease serve as evidence that prostate cancers can progress by circumventing T cell immune surveillance [17, 18]. For these reasons, and given that the prostate is an expendable organ and many tissue-specific proteins are already known, there has been much exploration of prostate-specific proteins as tumor vaccine antigens [19, 20]. In addition to dendritic cell-based vaccines, including sipuleucel-T, other vaccine strategies that have been evaluated include the use of whole tumor cells (GVAX) [21], recombinant viral vectors (PSA-TRICOM, PROSTVAC) [22], DNA (pTVG-HP) [23, 24], and purified proteins or peptides. Additional immunotherapy strategies in clinical trials in metastatic prostate cancer include the evaluation of checkpoint inhibitors to enhance activation of anti-tumor T cell response [21, 25–27]. Among the agents currently in clinical trials are those directed against cytotoxic T-lymphocyte associated protein-4 (CTLA-4), programmed cell death 1 (PD-1) and its ligands, and lymphocyte activation gene-3 (LAG-3).

Sipuleucel-T is currently the only approved immunotherapy approach for mCRPC and was shown to produce a survival advantage compared to placebo in a pivotal phase III randomized, placebo-controlled clinical trial. Consistent survival findings were also reported in two smaller randomized placebo controlled studies that were not powered for overall survival (OS) as the primary end point [9–11, 28]. It is a cancer vaccine derived from a recombinant fusion protein of prostatic acid phosphatase (PAP) and granulocyte-macrophage colony-stimulating factor (GM-CSF) that is used to activate autologous antigen-presenting cells (APCs) [19]. Treatment with vaccines, such as sipuleucel-T, is thought to induce tumor-specific immune responses and long-surviving memory T cells that potentially may continue to have anti-tumor effects long after it is administered [19, 20].

Other organizations, both U.S.-based and international, have developed guidelines concerning the clinical management of prostate cancer. Sipuleucel-T is currently the only immunotherapeutic agent approved by the U.S. Food and Drug Administration (FDA) and the European Medicines Agency (EMA) for prostate cancer. Thus, the National Comprehensive Cancer Network (NCCN), American Urological Association (AUA), American Society of Clinical Oncology (ASCO), and European Association of Urology (EAU) discuss sipuleucel-T as a treatment option for patients with mildly symptomatic or asymptomatic mCRCP and provide details of its approval based on improvement in OS [29–32]. However, due to differences in the international healthcare funding structure, guidance from the National Institute for Health and Care Excellence (NICE) does not recommend its use based on its incremental cost-effectiveness ration (ICER) vs. best standard care [33]. Although there is guidance for its use based on its approved indication, there is no consensus provided on sequencing with other therapies, monitoring response during treatment, and determining when to begin subsequent treatment. Thus, this consensus statement was developed to provide consensuses where current guidance is lacking for cancer immunotherapy agents, specifically for sipuleceul-T in this iteration. In addition, these guidelines provide information on future perspectives such as combination approaches and other immunotherapy agents in development, with plans to update these recommendations as further immunotherapeutic agents become approved in this disease setting.

SITC is a non-profit organization dedicated to advancing the science and application of cancer immunotherapy with the goal to improve outcomes for people with cancer. In order to provide guidance for practicing clinicians, SITC has established disease-specific panels to address the application of immunotherapy in the clinical setting and generate consensus guidelines. The Prostate Cancer Immunotherapy Guidelines panel, consisting of U.S. based physicians, nurses, and patient advocates, met in October 2014 to address the currently approved as well as emerging immunotherapies for prostate cancer. The discussion of this panel meeting focused on issues related to patient selection, monitoring of patients during and after treatment, sequencing of treatment with other available therapies, and any special issues for consideration, with the goal to generate a consensus statement on the clinical use of immunotherapy for prostate cancer patients. Moreover, a systematic literature search and review was performed to identify and evaluate the current evidence concerning the role of immunotherapy for prostate cancer. The overall goal of this consensus paper is to provide guidance for the clinical application of immunotherapy in prostate cancer patients and to provide the foundation to include future therapies with updates to these guidelines as warranted in an ever-changing therapeutic landscape.

## Methods

### Consensus statement policy

This consensus statement was prepared using the Institute of Medicine’s March 2011 Standards for Developing Trustworthy Clinical Practice Guidelines [34]. In addition, the previously released SITC consensus guidelines were used as a model to develop and organize this manuscript as previously described [35]. To develop these guidelines, SITC convened a panel led by a steering committee of prostate cancer experts to meet in October 2014 with the goal to develop clinical treatment guidelines for immunotherapy in prostate cancer patients. This consensus statement is only intended to provide guidance. It is not to be used as a substitute for the individual professional judgment of the treating physician. The full version of this consensus report and others can be found on the SITC website [36]. Because of differences in drug approval, availability, and regulations in other countries, the panel focused on drugs currently approved by U.S. Food and Drug Administration (FDA) for the treatment of patients in the United States. Given this, the consensus panel was U.S. based, and discussion focused on issues related to U.S. based clinical practice.

### Consensus panel and conflicts of interest

Following the methods used for the previous SITC consensus guidelines, panel members were both SITC members and nonmembers consisting of multidisciplinary experts encompassing clinicians and populations expected to be affected by the development of recommendations. All panel members were required to disclose any conflicts of interest using the SITC disclosure form, which requires full financial and other disclosures concerning relationships with commercial entities that could be expected to have direct regulatory or commercial impact resulting from the publication of this statement. No commercial funding was provided to support the consensus panel, literature review, or the preparation of this manuscript.

The consensus panel, consisting of 21 participants, including 14 medical oncologists, 3 urologists, 1 FDA physician-representative, 1 expert in translational research, 1 urologic oncology nurse, and 1 patient advocate, met in October 2014 (Additional file 1). In this meeting, results were reviewed from a previously distributed questionnaire to collect information regarding the panel member’s role in the care of prostate cancer patients, primary clinical focus, experience with FDA-approved agents for prostate cancer, and current practices for the use or recommended use of such agents (Additional file 2). The final version of this consensus statement was posted for an open comment period to the entire SITC membership (Additional file 3).

### Literature review

The MEDLINE database was used to perform the literature search using the terms “sipuleucel-T,” “prostate cancer and ipilimumab,” “prostate cancer and vaccine,” “prostate cancer and immunotherapy,” and “prostate cancer and therapeutic vaccine.” The search was limited to include clinical trials, meta-analyses, practice guidelines, and research in humans. The search, conducted on September 29, 2014, encompassed articles published 2006–2014. Phase I and phase I/II trials, as well as review articles, were excluded from the literature search. However, key early reports, meta-analyses, and guideline reports entered the panel discussion. After removing duplicates, reviewing the references for accuracy, and supplementing with additional references as identified by the consensus panel a 34-item bibliography was finalized (Additional file 4). Using the previously established grading system [28], the supporting literature was graded into three levels. To summarize, Level A was defined as strong, evidence-based data derived from prospective, randomized clinical trials and meta-analyses. Level B literature consisted of moderately supported data from uncontrolled, prospective clinical trials. Level C represented weak supporting data derived from reviews and case reports.

## Consensus recommendations

### Immunotherapy for non-mCRPC

#### Is there a role for the use of FDA-approved immunotherapy in patients with prostate cancer with non-metastatic, non-castrate disease?

There was uniformity of opinion that there is no FDA-approved immunotherapy agent for patients with prostate cancer without metastases, whether castration-sensitive or castration-resistant. Similarly, there was uniformity of opinion that the only immunotherapy agent currently approved by the FDA for the treatment of prostate cancer is sipuleucel-T, which is indicated for patients with asymptomatic or minimally symptomatic mCRPC. Considerable discussion ensued, however, regarding the potential for immunotherapy in an earlier patient disease setting in which immune responsiveness may be greater. It was generally believed that clinical trials of immunotherapy should be pursued in earlier disease states with appropriate immune monitoring.

#### Literature review and analysis

Sipuleucel-T is approved for mCRPC, and it is noteworthy that it is used in asymptomatic or minimally symptomatic patients where there was a survival benefit compared to the control group. Furthermore, additional retrospective analysis reported an association with a lower baseline serum PSA at the start of treatment with greater OS benefit from sipuleucel-T [37]. Analysis of immune parameters that correlated with survival in the phase III trials demonstrated that the activation and the number of activated APCs in the administered product correlated with longer survival [28]. This is interpreted to be associated with the development of a long-term immune response, potentially leading to prolonged OS [28]. There have been some studies of sipuleucel-T in patients with non-metastatic disease, including a randomized study, suggesting an improvement in PSA doubling after testosterone normalization following limited ADT in vaccine vs. placebo-treated patients [38].

Data from a trial with another immunological agent have similarly suggested a possible benefit in patients with lower disease burden. A recently published study of ipilimumab in patients with mCRPC who were treated after palliative radiation and had progressed after docetaxel did not meet its primary endpoint target of increased OS [25]. However, in a retrospective subgroup analysis, this study suggested that a subpopulation with less advanced disease derived greater benefit from ipilimumab compared to placebo [25]. This subgroup consisted of patients with non-visceral disease, alkaline phosphatase less than 1.5 times the upper limit of normal, and hemoglobin of 11 gm/dL or greater. For this subset, the median OS of patients treated with ipilimumab was 22.7 months compared with 15.8 months for the patients who received placebo. The median OS for patients with even one of the poor prognostic factors listed above was 6.5 months among those treated with ipilimumab and 7.3 months for those who received placebo (*p* = 0.8756). However, we would underscore that to date there are no prospective data to support the use of CTLA-4 as a monotherapy for mCRPC. Similarly, in two phase I trials of nivolumab, an anti-PD-1 antibody, among 25 heavily pre-treated patients with prostate cancer, there were no objective responses [26, 27]. Consequently, there are also no data to support the efficacy of checkpoint blockade with PD-1 or PD-L1 blockade as monotherapy in patients with advanced prostate cancer.

#### Consensus recommendations

Based on recent data and accumulated experience with immune activating agents in patients with prostate cancer, immunotherapy may achieve greater benefit among mCPRC patients treated earlier in the disease course. The level of data supporting this is Level B from subset analyses of randomized clinical trials in patients with mCRPC [9–11, 25]. However, there are currently no appropriate efficacy data to support the use of sipuleucel-T in patients with non-metastatic prostate cancer.

## Immunotherapy for mCRPC

### What is the appropriate use of immunotherapy in the treatment of mCRPC?

The panel was in agreement that there is a role for the use of sipuleucel-T in the management of mCRPC prior to chemotherapy in the era of abiraterone and enzalutamide. The role of sipuleucel-T may be somewhat limited, but the optimal patients for this approach should be carefully defined, such that patients with mCRPC have as many options as possible. Its use in an earlier disease state may theoretically be more optimal as discussed above, given that a retrospective evaluation showed that a lower PSA level at the start of treatment appeared to correlate with longer OS in the IMPACT trial [37]. However, the current evidence-based recommendation is in asymptomatic or minimally symptomatic patients with mCRPC. This recommendation could be more stringently defined to optimize benefit. This recommendation is supported by Level A evidence from randomized trials and meta-analyses [9–11, 39].

#### Literature review and analysis

The pivotal placebo-controlled phase III trial that led to the approval of sipuleucel-T, and two other supportive phase III trials, showed a clinically meaningful and statistically significant survival benefit (25.8 months versus 21.7 months, *p* = 0.03, hazard ratio 0.78), independent of the outcome of PSA decline or progression-free survival (PFS) [9–11]. There was some conjecture that in the setting of immunotherapy, the short term response parameters may not be surrogates for OS. Reports and analysis suggest that immune response generated by sipuleucel-T may correlate with survival benefit [28, 40, 41].

#### Consensus recommendations

The level of the evidence to support sipuleucel-T in mCRPC was debated by the panel. Those who based their position on the three randomized trials felt the data was level A in support of sipuleucel-T, based on an appropriately powered randomized controlled study and two meta-analyses of the clinical trials studies confirming a statistically significant and clinically meaningful survival benefit [30, 31]. Others felt the evidence to be weak to moderate given that one of the supportive trials was not completed, and OS was not the primary endpoint of two of these trials. It was noted that the total number of patients and statistical power were less than required by the AUA Guidelines. Therefore, AUA Guidelines recently considered the evidence to be level B [32]. In addition, guidelines from ASCO rated the recommendation strength for sipuleucel-T to be weak due to unclear quality of life benefit, although benefits in overall survival are supported [31]. However, the NCCN guidelines rated the strength of the recommendations of sipuleucel-T in this setting as category 1 [29]. Moreover, guidance from the EAU also rated the recommendation to use sipuleucel T in this setting as level A [30], consistent with the SITC rating system in which the evidence was considered level A.

This difference of opinion may account for the perceived need for additional investigations into the mechanism of action, and the investigations into other measures of immune activation, resulting from this treatment as well as other immunotherapies entering clinical evaluation. With sipuleucel-T, there may have been a long-term immune activation in those who appeared to have a longer OS [28], and others have described an alteration in the tumor growth kinetics, and changes in tumor microenvironment [42, 43]. Because a vaccine-induced immune response should lead to initial immune-mediated tumor cell death, other antigens within the tumor (e.g., neo-epitopes) can theoretically be presented back to the immune system. This tumor immunity cycle can lead to a broader, and potentially more clinically relevant, immune response known as “antigen spread.” However, this is an ongoing dynamic and iterative process that may take some time before becoming clinically apparent. This process of antigen spreading has been demonstrated following treatment with sipuleucel-T and retrospectively demonstrated to be associated with prolonged OS [41]. This process needs to be further evaluated as additional immunotherapies enter the clinical arena.

With respect to an overall role for immunotherapy in the treatment of mCRPC, the general consensus was that there are major characteristics of mCRPC that favor an immunotherapy approach. Therefore, the goals should be to define optimal patient and tumor characteristics, identify best immunotherapy approaches, and identify the optimal sequence of immunotherapy with other available treatments that will benefit patients. Additional discussion centered on the type of clinical endpoints that reflect clinical benefit.

### Can optimal patient candidates be identified for an immunotherapy approach? will it be the same or different for different immunotherapy agents?

With sipuleucel-T, the recommendation was for use in asymptomatic or minimally symptomatic patients with mCRPC. Further retrospective analysis defined those with lower PSA to have a potentially greater survival benefit [37]. In the subgroup analysis in the randomized trial of ipilimumab versus placebo, the patients who did not have poor prognostic features (no visceral metastases, hemoglobin > 11, alkaline phosphatase < 1.5 ULN) had greater survival with ipilimumab treatment compared with those treated with placebo [25], suggesting that similar populations may be preferable for other immunotherapy agents.

#### Consensus recommendations

The majority of the panel (71%) recommended using clinical laboratory tests to select patients for use of sipuleucel-T by evaluating PSA, complete blood count (CBC), and liver enzymes. The intent was to rule out individuals at risk for rapid disease progression, although no specific laboratory thresholds precluding patients from treatment were discussed. In addition, the panel also discussed whether the extent of disease by imaging determines whether to initiate treatment with sipuleucel-T. Sixty-four percent of the panel felt that the extent of disease as determined by imaging should be used to select patients for sipuleucel-T. Overall, the panel recommended using the rate of change at disease sites by imaging, reflecting the pace of the disease, to determine whether immunotherapy is appropriate. The panel recommended that those with rapidly growing disease not receive immunotherapy. Similarly, the panel would exclude patients with liver metastases.

These recommendations were based on prospective analysis of stratification factors and retrospective analysis of clinical and laboratory factors among patients enrolled in prospective randomized clinical trials of sipuleucel-T.

#### Literature review and analysis

Based on data from the randomized trials of sipuleucel-T, those patients with minimally to asymptomatic disease and low initial PSA had the greatest survival benefit, reflecting earlier and less rapidly progressive disease [9–11, 37]. Additionally those able to mount an immune response also appeared to have greater benefit [28]. The subgroup analysis from the randomized trial of ipilimumab versus placebo also supports the selection of patients with better prognostic features as those able to generate an immune response and derive benefit from immunologic treatment.

Clinical parameters can be selected prior to treatment, but currently there are no biomarkers that will predict the expected degree of immunologic activation. The discovery of reliable predictive immune biomarkers remains a high research priority.

### What is the impact of corticosteroid therapy (used in conjunction with previous treatment), chemotherapy, and secondary hormonal agents? Can patients continue on corticosteroids and receive immunotherapy for mCRPC, in particular sipuleucel-T, if utilized following these agents?

Corticosteroids are included in regimens used in the initial treatment of metastatic prostate cancer, and may precede treatment with sipuleucel-T, and possibly other evolving immunotherapies. The duration of such therapy will impact whether there needs to be a weaning process, or whether patients will require continued physiologic corticosteroid therapy. However, the prior use of high dose corticosteroids is not thought to be problematic for subsequent immune-based treatment.

#### Consensus recommendations

The discussion evaluated both stopping steroids and continuing physiologic doses. Essentially, it was felt that after a short course of corticosteroids, it is not necessary to wean off corticosteroids. The panel was in agreement (100%) that it is not necessary to wean corticosteroids from a dose equivalent of 10 mg/day prednisone in order to treat with sipuleucel-T.

For patients who have been treated with abiraterone/corticosteroids for 6 months or longer, and are taking physiologic doses of glucocorticoids, the patient can proceed with sipuleucel-T and would be expected to produce adequate numbers of dendritic cells. The level of evidence is considered Level B, based on a randomized phase II trial of concurrent vs. sequential abiraterone and sipuleucel-T showing no impact of abiraterone/corticosteroids on sipuleucel-T induced APC activation and antigen spread [44].

#### Literature review and analysis

Several investigators have evaluated the immune response to sipuleucel-T and the enumeration of activated APCs, which is an FDA-approved release criteria for this product [45]. Whereas the number and activation state of APCs produced may impact the outcome of treatment [28], ongoing corticosteroid treatment as used in prostate cancer does not appear to affect the level of activation of APCs or subsequent antigen spread [39], both of which have been positively associated with clinical outcome [25, 32, 33]. There is no data suggesting an impact of corticosteroids on the clinical outcome of treatment with sipuleucel-T [44–47].

### What is the preferred sequence of agents for the management of patients with minimally symptomatic (or asymptomatic) metastatic, castration-resistant prostate cancer?

Several agents have been approved for second-line treatment of prostate cancer, once the disease has become refractory to initial androgen deprivation. Sipuleucel-T is approved for minimally symptomatic or asymptomatic patients in this setting. Investigational immunotherapies are also being evaluated in this setting. The rationale for deciding which agent to use first with disease recurrence after initial ADT currently depends on the clinical status of the patient and the extent, site(s), and pace of the disease.

#### Consensus recommendations

Two approaches were discussed by the panel as follows: 1) sipuleucel-T first or 2) an androgen receptor-targeted agent (such as abiraterone acetate or enzalutamide) first followed by sipuleucel-T. The majority of the panel recommended the use of sipuleucel-T first (90%), while the minority of the panel recommended the second approach (10%). Moreover, the panel was in agreement (100%) that it was optimal to use one of these approaches prior to radium and chemotherapy. Given the importance of immunologic activation and the asymptomatic status of the patient, 100% of the panel recommended that when sipuleucel-T is used, it be used first if all other criteria are met.

#### Literature review and analysis

As noted in the above discussions and literature review, the primary goal of sipuleucel-T therapy is to generate an appropriate immune response directed against the prostate tumor [28, 41, 43, 46–48]. This immune response, once generated, can persist long after the treatment is given, unlike the expected impact of an androgen receptor targeted therapy. A number of phase I-II clinical trials are underway to evaluate the optimal sequencing of sipuleucel-T with other agents and to investigate whether measures of immunological activation correlate with clinical outcome.

### What are the special issues and clinical management recommendations in the use of sipuleucel-T for the treatment of mCRPC?

There are detailed guidelines in the pharmaceutical package insert for the management of patients undergoing treatment with sipuleucel-T. The panel discussed these guidelines in detail as well as the issues related to patient monitoring. The relevant issues discussed included the evaluation of hematologic parameters for apheresis, monitoring patients during the infusion of activated cells, issues of central line infection, and follow-up monitoring of disease status after treatment is completed.

#### Consensus recommendations

The panel accepted the guidelines as outlined in the pharmaceutical Full Prescribing Information (package insert) for the production and administration of this specific immunotherapy agent. Any guidelines regarding other immunotherapy will be product specific. It was recommended that laboratory parameters as noted in the Full Prescribing Information through the course of apheresis be followed. Guidelines for hematologic parameters for apheresis are determined by the apheresis center. It was generally believed by the panel that patients do not require clinical evaluation prior to each cellular infusion if the previous one was uncomplicated. Infusions are typically completed in oncology or urology infusion centers and are monitored as per any other cellular infusion [9–11].

#### Literature review and analysis

The major concerns are related to the need in some patients for central venous access to accomplish apheresis and the need to maintain such a line for the 4-6 weeks required for the procedures. This was evaluated for the IMPACT trial in which 23% of subjects required a central line for apheresis, and 12% developed infection related to catheter use [9–11]. However, as centers become more experienced management of central lines improves in general. Peripheral veins can be used in subjects as well, depending on the availability and quality of peripheral veins as determined by the apheresis center.

### What are the monitoring parameters following sipuleucel-T therapy?

#### Consensus recommendations

Standard practice is used in terms of PSA and radiologic monitoring for patients with advanced prostate cancer. Eighty percent of the panel stated they would not change their standard monitoring procedure, while 20% reported that they would by obtaining a new baseline status immediately after completion of the infusions. These recommendations are based on level C evidence, as the evaluations performed in clinical trials leading to the approval of sipuleucel-T were typically done at 12 weeks rather than immediately following treatment.

#### Literature review and analysis

There are currently no additional monitoring procedures or biomarkers for following patients treated with sipuleucel-T [8–10]. The apheresis product is assessed for the number of CD54 cells, but this is not followed after completion of the infusions [45].

Recent papers suggest that eosinophilia may correlate with prolonged survival in patients receiving sipuleucel-T for mCRPC, but this is not yet standard and deserves further investigation [49].

### How long do you wait after the last biweekly treatment with sipuleucel-T before considering another therapy?

#### Consensus recommendations

Although there was not a consensus of opinion among the panel, several options were discussed. The minority of the panel (10%) recommended moving to a new therapy immediately. However, this option was recommended in the setting of a program that is a two-part treatment approach (e.g., sipuleucel-T followed by enzalutamide). Thirty percent of the panel recommended that it should depend on individual patient characteristics and the pace of the disease. The majority of the panel (60%) recommended waiting for an event/progression before beginning a subsequent therapy.

### How do you determine that it is time to start another treatment after having used sipuleucel-T?

#### Consensus recommendations

The next treatment may be triggered by an event or initiated earlier. Patients must be aware that immunologic therapy may take time and that typical measures of response, as determined by decreases in serum PSA or size of lesions on radiographic studies, are not likely to be affected. To address the issues involved with the atypical response measured with immunotherapy, the FDA has drafted guidance for industry concerning treatment past progression when using therapeutic cancer vaccines [50]. Moreover, in measuring treatment response, immune-related response criteria have been developed to more accurately measure the response patterns observed with immunotherapy [51]. Managing patient expectations with a therapy such as this is consequently important and complex. These outcomes are based on results from phase III trials and on clinical experience, and hence are considered Level A evidence.

### Are there other considerations for repeat dosing or changes in the dosing schedule of sipuleucel-T?

#### Consensus recommendations

There are clinical situations in which there may be increased intervals between doses. There are no immunological hypotheses that would preclude continuing infusions, even with a delay. Currently the data that exist are observations during the randomized clinical trials.

#### Literature review and analysis

The recommendations from this discussion were based on the results and data from the phase III trials and meta-analyses [9–11, 39, 52]. Essentially they reflect the methodology in the literature and the pharmaceutical guidelines. At this time, there are no data to recommend moving the frequency from every 2 weeks to every 4 weeks. However, there is no recognized harm from a delay if it occurs.

### Cost and value of sipuleucel-T

As the rising cost of cancer care has increasingly become an area of concern among the oncology community, a brief overview of the cost as well as considerations of the value of sipuleucel-T was added after the panel meeting. The average wholesale price for sipuleucel-T is approximately $93,000 per patient for a complete course of treatment (over about 1 month). While this is considerably higher as a monthly cost compared with other treatments for advanced prostate cancer, this cost is similar to costs for other therapies when factored over time (Table 1). Moreover, a direct cost comparison does not take into account the relative paucity of side effects from sipuleucel-T compared with chemotherapies that may lead to additional costs due to hospitalization, cost of growth factor support, cost of multiple infusions, and less tangible costs due to loss of work from multiple treatment visits [53].

**Table 1:** Estimated prices of agents approved to treat prostate cancer

Treatment	Cost of treatment alone^a^	Median overall survival benefit
sipuleucel-T	$93,000 (median 3 cycles)	25.8 months vs. 21.7 months [9]
enzalutamide	$89,952 (median of 8 cycles)	18.4 months vs. 13.6 months [4]
abiraterone	$144,950 (median of 14 cycles)	34.7 vs. 30.3 months [6, 59]
docetaxel	$25,000 (median of 10 cycles)	18.9 months vs. 16.5 months [60]
cabazitaxel	$68,751 (median of 6 cycles)	15.1 months vs. 12.7 months [7]
radium-223	$155,048 (median of 6 injections)	14.9 months vs. 11.3 months [8]

^a^As determined by using estimates of average wholesale drug prices retrieved from UptoDate accessed 11/17/2016

## Future perspectives

### What is the potential use of sipuleucel-T in combination with other agents?

The panel discussed the potential of combining sipuleucel-T with other agents. Agents discussed included androgen pathway targeted agents (e.g., bicalutamide, nilutamide, enzalutamide, and abiraterone acetate), zoledronic acid, and denosumab. The majority of the panel (58%) reported having ever combined sipuleucel-T with other agents. However, the panel was in agreement (100%) that they do not routinely use a combination approach with sipuleucel-T. The consensus discussion continued with considering whether sipuleucel-T should be used in combination, and concluded that all of these therapies were reasonable to investigate in combination with sipuleucel-T. In fact, a number of phase I and phase II trials are active or in development [44, 47, 48] (Table 2). This assessment is considered Level B evidence, based on numerous ongoing prospective clinical trials.

**Table 2:** Examples of ongoing sipuleucel-T combination studies

Trial	National clinical trial identifier	Status^a^
Concurrent vs. sequential sipuleucel-T and abiraterone	NCT01487863	Randomized phase II, Reported in 2015 [44]
Sipuleucel-T +/- RT	NCT01807065	Randomized Phase II, Recruiting, Expected completion date June 2017
Sipuleucel-T +/- pTVG-HP DNA booster vaccine	NCT01706458	Randomized pilot study, Recruiting, Expected completion date December 2016
Sipuleucel-T + indoximod	NCT01560923	Randomized phase II Recruiting, Expected completion date December 2016
Sipuleucel-T +/- ipilimumab	NCT01804465	Randomized phase II, Recruiting, Expected completion date December 2017
Sipuleucel-T +/- radium - 223	NCT02463799	Randomized phase II, Recruiting, Expected completion date December 2018

^a^ As determined by ClinicalTrials.gov accessed October 31, 2016

The panel also discussed these issues in the context of immunotherapy agents that are in very advanced stages of clinical evaluation. PSA-TRICOM is a therapeutic vaccine associated with a 44% reduction of risk of death in a randomized phase II trial [22]. A subsequent phase III study completed enrollment with 1297 patients in 2015, with overall survival expected to be reported in 2017. Recent reports describe the outcome of two cooperative group clinical trials with combinations of PSA-TRICOM vaccine with docetaxel or with sequential androgen ablation therapy [54, 55]. Immunotherapy trials combining vaccines and checkpoint inhibitors to further activate the immune response are also of interest and ongoing [21, 56, 57]. In addition, the use of other agents to enhance antigen presentation is being explored. Many different combination approaches, with sipuleucel-T and with other immunotherapy agents, are ongoing [58]. Phase III trials of vaccines and immune modulators are ongoing (Table 3). Immune monitoring continues to be a major component of evaluating the effects of immunotherapy.

**Table 3:** Ongoing phase III immunotherapy trials in mCRPC

Trial	National clinical trial identifier	Status*
PSA-TRICOM +/- GM-CSF	NCT01322490	Ongoing, Expected completion date June 2017
Tasquinimod	NCT01234311	Completed, Reported in 2016 [61]
Ipilimumab in chemo-naïve mCRPC	NCT01057810	Completed, Reported in 2016 [38]

* As determined by ClinicalTrials.gov accessed October 31, 2016

## Conclusions

Currently there is one FDA-approved immunotherapeutic agent for the treatment of mCRPC, providing a proof of principle that continues to stimulate the investigation of other immune approaches for prostate cancer. Additional phase III trials of immunotherapy agents as well as combination trials are ongoing and results will be reported in the near future. Further elucidation of patient-related characteristics and predictive immunological parameters of clinical benefit is the subject of ongoing investigations. The key to future development of immunotherapy in prostate cancer will be the delineation of the optimal sequence of immunotherapy, as single agents or in combination, with other active therapies for this disease.

## Additional files

Additional file 1: Cancer Immunotherapy Guidelines Prostate Cancer Roster. (DOCX 12 kb)
Additional file 2: Pre-meeting Survey Questions and Responses. (DOCX 30 kb)
Additional file 3: Comments Recieved during Review. (DOCX 12 kb)
Additional file 4: Annotated Bibliography. (PDF 231 kb)

## Abbreviations

ADT: Androgen deprivation therapy
APC: Antigen-presenting cell
ASCO: American Society of Clinical Oncology
AUA: American Urologic Association
CBC: Complete blood count
CTLA-4: Cytotoxic T-lymphocyte associated protein-4
EAU: European Association of Urology
FDA: U.S. Food and Drug Administration
GM-CSF: Granulocyte-macrophage colony-stimulating factor
ICER: Incremental cost effectiveness ratio
LAG-3: Lymphocyte activation gene-3
mCRPC: Metastatic castrate resistant prostate cancer
NCCN: National Comprehensive Cancer Network
NICE: National Institute for Health and Care Excellence
OS: Overall survival
PAP: Prostatic acid phosphatase
PD-1: Programmed cell death 1
PD-L1: Programmed cell death-ligand 1
PFS: Progression-free survival
SITC: Society for Immunotherapy of Cancer

## References

[1] Siegel RL, Miller KD, Jemal A (2016). Cancer statistics, 2016. CA Cancer J Clin.

[2] Siegel RL, Miller KD, Jemal A (2015). Cancer statistics, 2015. CA Cancer J Clin.

[3] Potosky AL, Miller BA, Albertsen PC, Kramer BS (1995). The role of increasing detection in the rising incidence of prostate cancer. JAMA.

[4] Scher HI, Fizazi K, Saad F, Taplin ME, Sternberg CN, Miller K (2012). Increased survival with enzalutamide in prostate cancer after chemotherapy. N Engl J Med.

[5] Fizazi K, Scher HI, Molina A, Logothetis CJ, Chi KN, Jones RJ (2012). Abiraterone acetate for treatment of metastatic castration-resistant prostate cancer: final overall survival analysis of the COU-AA-301 randomised, double-blind, placebo-controlled phase 3 study. Lancet Oncol.

[6] Ryan CJ, Smith MR, de Bono JS, Molina A, Logothetis CJ, de Souza P (2013). Abiraterone in metastatic prostate cancer without previous chemotherapy. N Engl J Med.

[7] de Bono JS, Oudard S, Ozguroglu M, Hansen S, Machiels JP, Kocak I (2010). Prednisone plus cabazitaxel or mitoxantrone for metastatic castration-resistant prostate cancer progressing after docetaxel treatment: a randomised open-label trial. Lancet.

[8] Parker C, Nilsson S, Heinrich D, Helle SI, O'Sullivan JM, Fossa SD (2013). Alpha emitter radium-223 and survival in metastatic prostate cancer. N Engl J Med.

[9] Kantoff PW, Higano CS, Shore ND, Berger ER, Small EJ, Penson DF (2010). Sipuleucel-T immunotherapy for castration-resistant prostate cancer. N Engl J Med.

[10] Small EJ, Schellhammer PF, Higano CS, Redfern CH, Nemunaitis JJ, Valone FH (2006). Placebo-controlled phase III trial of immunologic therapy with sipuleucel-T (APC8015) in patients with metastatic, asymptomatic hormone refractory prostate cancer. J Clin Oncol.

[11] Higano CS, Schellhammer PF, Small EJ, Burch PA, Nemunaitis J, Yuh L (2009). Integrated data from 2 randomized, double-blind, placebo-controlled, phase 3 trials of active cellular immunotherapy with sipuleucel-T in advanced prostate cancer. Cancer.

[12] Gannon PO, Poisson AO, Delvoye N, Lapointe R, Mes-Masson AM, Saad F (2009). Characterization of the intra-prostatic immune cell infiltration in androgen-deprived prostate cancer patients. J Immunol Methods.

[13] Sfanos KS, Bruno TC, Meeker AK, De Marzo AM, Isaacs WB, Drake CG (2009). Human prostate-infiltrating CD8+ T lymphocytes are oligoclonal and PD-1+. Prostate.

[14] Mercader M, Bodner BK, Moser MT, Kwon PS, Park ES, Manecke RG (2001). T cell infiltration of the prostate induced by androgen withdrawal in patients with prostate cancer. Proc Natl Acad Sci U S A.

[15] McNeel DG, Nguyen LD, Ellis WJ, Higano CS, Lange PH, Disis ML (2001). Naturally occurring prostate cancer antigen-specific T cell responses of a Th1 phenotype can be detected in patients with prostate cancer. Prostate.

[16] Olson BM, McNeel DG (2007). Antibody and T-cell responses specific for the androgen receptor in patients with prostate cancer. Prostate.

[17] Sanda MG, Restifo NP, Walsh JC, Kawakami Y, Nelson WG, Pardoll DM (1995). Molecular characterization of defective antigen processing in human prostate cancer. J Natl Cancer Inst.

[18] Bander NH, Yao D, Liu H, Chen YT, Steiner M, Zuccaro W (1997). MHC class I and II expression in prostate carcinoma and modulation by interferon-alpha and -gamma. Prostate.

[19] Gulley JL, Madan RA, Heery CR. Therapeutic vaccines and immunotherapy in castration-resistant prostate cancer: current progress and clinical applications. Am Soc Clin Oncol Educ Book. 2013. doi:10.1200/EdBook_AM.2013.33.e166. 10.1200/EdBook_AM.2013.33.e166 PMC6594370 23714490

[20] Slovin SF. Immunotherapeutic approaches in prostate cancer: combinations and clinical integration. Am Soc Clin Oncol Educ Book. 2015:e275-83. doi:10.14694/EdBook_AM.2015.35.e275 10.14694/EdBook_AM.2015.35.e275 25993186

[21] van den Eertwegh AJ, Versluis J, van den Berg HP, Santegoets SJ, van Moorselaar RJ, van der Sluis TM (2012). Combined immunotherapy with granulocyte-macrophage colony-stimulating factor-transduced allogeneic prostate cancer cells and ipilimumab in patients with metastatic castration-resistant prostate cancer: a phase 1 dose-escalation trial. Lancet Oncol.

[22] Kantoff PW, Schuetz TJ, Blumenstein BA, Glode LM, Bilhartz DL, Wyand M (2010). Overall survival analysis of a phase II randomized controlled trial of a Poxviral-based PSA-targeted immunotherapy in metastatic castration-resistant prostate cancer. J Clin Oncol.

[23] McNeel DG, Dunphy EJ, Davies JG, Frye TP, Johnson LE, Staab MJ (2009). Safety and immunological efficacy of a DNA vaccine encoding prostatic acid phosphatase in patients with stage D0 prostate cancer. J Clin Oncol.

[24] Eriksson F, Totterman T, Maltais AK, Pisa P, Yachnin J (2013). DNA vaccine coding for the rhesus prostate specific antigen delivered by intradermal electroporation in patients with relapsed prostate cancer. Vaccine.

[25] Kwon ED, Drake CG, Scher HI, Fizazi K, Bossi A, van den Eertwegh AJ (2014). Ipilimumab versus placebo after radiotherapy in patients with metastatic castration-resistant prostate cancer that had progressed after docetaxel chemotherapy (CA184-043): a multicentre, randomised, double-blind, phase 3 trial. Lancet Oncol.

[26] Topalian SL, Hodi FS, Brahmer JR, Gettinger SN, Smith DC, McDermott DF (2012). Safety, activity, and immune correlates of anti-PD-1 antibody in cancer. N Engl J Med.

[27] Brahmer JR, Drake CG, Wollner I, Powderly JD, Picus J, Sharfman WH (2010). Phase I study of single-agent anti-programmed death-1 (MDX-1106) in refractory solid tumors: safety, clinical activity, pharmacodynamics, and immunologic correlates. J Clin Oncol.

[28] Sheikh NA, Petrylak D, Kantoff PW, Dela Rosa C, Stewart FP, Kuan LY (2013). Sipuleucel-T immune parameters correlate with survival: an analysis of the randomized phase 3 clinical trials in men with castration-resistant prostate cancer. Cancer Immunol Immunother.

[29] National Comprehensive Cancer Network. Prostate Cancer (Version 3.2016). https://www.nccn.org/professionals/physician_gls/pdf/prostate.pdf. Accessed 24 Oct 2016.

[30] European Association of Urology. Prostate Cancer. http://uroweb.org/guideline/prostate-cancer/. Accessed 24 Oct 2016.

[31] Basch E, Loblaw DA, Oliver TK, Carducci M, Chen RC, Frame JN (2014). Systemic therapy in men with metastatic castration-resistant prostate cancer: American society of clinical oncology and cancer care Ontario clinical practice guideline. J Clin Oncol.

[32] Lowrance WT, Roth BJ, Kirkby E, Murad MH, Cookson MS. Castration-Resistant Prostate Cancer: AUA Guideline Amendment 2015. J Urol. 2015. doi:10.1016/j.juro.2015.10.086. 10.1016/j.juro.2015.10.086 26498056

[33] Simpson EL, Davis S, Thokala P, Breeze PR, Bryden P, Wong R (2015). Sipuleucel-T for the treatment of metastatic hormone-relapsed prostate cancer: a nice single technology appraisal; an evidence review group perspective. Pharmacoecon.

[34] Institute of Medicine Committee on Standards for Developing Trustworthy Clinical Practice Guidelines. Graham R, Mancher M, Miller Wolman D, Greenfield S, Steinberg E, editors. Clinical Practice Guidelines We Can Trust. Washington (DC): National Academies Press (US); 2011. https://www.ncbi.nlm.nih.gov/pubmed/24983061. 24983061

[35] Kaufman HL, Kirkwood JM, Hodi FS, Agarwala S, Amatruda T, Bines SD (2013). The society for immunotherapy of cancer consensus statement on tumour immunotherapy for the treatment of cutaneous melanoma. Nat Rev Clin Oncol.

[36] Society for Immunotheray of Cancer (SITC). Cancer Immunotherapy Guidelines. 2013. http://www.sitcancer.org/about-sitc/initiatives/cancer-immunotherapy-guidelines. Accessed 04 Aug 2016.

[37] Schellhammer PF, Chodak G, Whitmore JB, Sims R, Frohlich MW, Kantoff PW (2013). Lower baseline prostate-specific antigen is associated with a greater overall survival benefit from sipuleucel-T in the immunotherapy for prostate adenocarcinoma treatment (IMPACT) trial. Urology.

[38] Beer TM, Bernstein GT, Corman JM, Glode LM, Hall SJ, Poll WL (2011). Randomized trial of autologous cellular immunotherapy with sipuleucel-T in androgen-dependent prostate cancer. Clin Cancer Res.

[39] Botrel TE, Clark O, Pompeo AC, Bretas FF, Sadi MV, Ferreira U (2012). Immunotherapy with Sipuleucel-T (APC8015) in patients with metastatic castration-refractory prostate cancer (mCRPC): a systematic review and meta-analysis. Int Braz J Urol.

[40] Mulders PF, De Santis M, Powles T, Fizazi K (2015). Targeted treatment of metastatic castration-resistant prostate cancer with sipuleucel-T immunotherapy. Cancer Immunol Immunother.

[41] GuhaThakurta D, Sheikh NA, Fan LQ, Kandadi H, Meagher TC, Hall SJ (2015). Humoral immune response against nontargeted tumor antigens after treatment with sipuleucel-T and its association with improved clinical outcome. Clin Cancer Res.

[42] Fong L, Carroll P, Weinberg V, Chan S, Lewis J, Corman J et al. Activated lymphocyte recruitment into the tumor microenvironment following preoperative sipuleucel-T for localized prostate cancer. J Natl Cancer Inst. 2014;106(11). doi:10.1093/jnci/dju268. 10.1093/jnci/dju268 PMC4241888 25255802

[43] Singh BH, Gulley JL (2014). Immunotherapy and therapeutic vaccines in prostate cancer: an update on current strategies and clinical implications. Asian J Androl.

[44] Small EJ, Lance RS, Gardner TA, Karsh LI, Fong L, McCoy C (2015). A randomized phase II trial of sipuleucel-T with concurrent versus sequential abiraterone acetate plus prednisone in metastatic castration-resistant prostate cancer. Clin Cancer Res.

[45] Sheikh NA, Jones LA (2008). CD54 is a surrogate marker of antigen presenting cell activation. Cancer Immunol Immunother.

[46] Thara E, Dorff TB, Averia-Suboc M, Luther M, Reed ME, Pinski JK (2012). Immune response to sipuleucel-T in prostate cancer. Cancers (Basel).

[47] Antonarakis E KA, Adams G, Karsh L, Elfiky A, Shore N. A randomized phase 2 study evaluating the optimal sequencing of sipuleucel-T and androgen deprivation therapy in biochemically-recurrent prostate cancer: Immune results with a focus on humoral responses. Proceedings of the European Association of Andrology Annual Congress, Stockholm. 2014;11-15 April(Poster 980).

[48] Quinn DI, Shore ND, Egawa S, Gerritsen WR, Fizazi K (2015). Immunotherapy for castration-resistant prostate cancer: Progress and new paradigms. Urol Oncol.

[49] McNeel DG, Gardner TA, Higano CS, Kantoff PW, Small EJ, Wener MH (2014). A transient increase in eosinophils is associated with prolonged survival in men with metastatic castration-resistant prostate cancer who receive sipuleucel-T. Cancer Immunol Res.

[50] US Food and Drug Administration. Guidance for Industry: Clinical Considerations for Therapeutic Cancer Vaccines. 2011. http://www.fda.gov/downloads/BiologicsBloodVaccines/GuidanceComplianceRegulatoryInformation/Guidances/Vaccines/UCM278673.pdf. Accessed 16 Nov 2016

[51] Wolchok JD, Hoos A, O'Day S, Weber JS, Hamid O, Lebbe C (2009). Guidelines for the evaluation of immune therapy activity in solid tumors: immune-related response criteria. Clin Cancer Res.

[52] Kawalec P, Paszulewicz A, Holko P, Pilc A (2012). Sipuleucel-T immunotherapy for castration-resistant prostate cancer. A systematic review and meta-analysis. Arch Med Sci.

[53] Fitch K, Pyenson B. Cancer patients recieving chemotherapy: opportunities for better management. Client Report. 2010;30:1-27.

[54] DiPaola RS, Chen YH, Bubley GJ, Stein MN, Hahn NM, Carducci MA (2015). A national multicenter phase 2 study of prostate-specific antigen (PSA) pox virus vaccine with sequential androgen ablation therapy in patients with PSA progression: ECOG 9802. Eur Urol.

[55] McNeel DG, Chen YH, Gulley JL, Dwyer AJ, Madan RA, Carducci MA (2015). Randomized phase II trial of docetaxel with or without PSA-TRICOM vaccine in patients with castrate-resistant metastatic prostate cancer: A trial of the ECOG-ACRIN cancer research group (E1809). Hum Vaccin Immunother.

[56] Karan D, Van Veldhuizen P (2012). Combination immunotherapy with prostate GVAX and ipilimumab: safety and toxicity. Immunotherapy.

[57] Jochems C, Tucker JA, Tsang KY, Madan RA, Dahut WL, Liewehr DJ (2014). A combination trial of vaccine plus ipilimumab in metastatic castration-resistant prostate cancer patients: immune correlates. Cancer Immunol Immunother.

[58] Gulley JL, Madan RA, Tsang KY, Jochems C, Marte JL, Farsaci B (2014). Immune impact induced by PROSTVAC (PSA-TRICOM), a therapeutic vaccine for prostate cancer. Cancer Immunol Res.

[59] Ryan CJ, Smith MR, Fizazi K, Saad F, Mulders PFA, Sternberg CN (2015). Abiraterone acetate plus prednisone versus placebo plus prednisone in chemotherapy-naive men with metastatic castration-resistant prostate cancer (COU-AA-302): final overall survival analysis of a randomised, double-blind, placebo-controlled phase 3 study. Lancet Oncol.

[60] Tannock IF, de Wit R, Berry WR, Horti J, Pluzanska A, Chi KN (2004). Docetaxel plus prednisone or mitoxantrone plus prednisone for advanced prostate cancer. N Engl J Med.

[61] Sternberg C, Armstrong A, Pili R, Ng S, Huddart R, Agarwal N (2016). Randomized, double-blind, placebo-controlled phase III study of tasquinimod in men with metastatic castration-resistant prostate cancer. J Clin Oncol.

