# Supplementary material for: The Society for Immunotherapy of Cancer consensus statement on immunotherapy for the treatment of prostate carcinoma
Source: J Immunother Cancer. 2016 Dec 20;4:92. doi: 10.1186/s40425-016-0198-x (PMC5170901; doi:10.1186/s40425-016-0198-x)
Supplement: Additional file 1: — Cancer Immunotherapy Guidelines Prostate Cancer Roster. (DOCX 12 kb) [file 40425_2016_198_MOESM1_ESM.docx]

**Appendix I: Participant List**

**Steering Committee:**

James Gulley, MD, PhD, FACP National Cancer Institute, National Institutes of Health

Douglas McNeel, MD, PhD University of Wisconsin, Madison

**Task Force Participants:**

Neil Bander, MD Weill Medical College of Cornell University

Tom Beer MD, FACP Oregon Health and Science University Knight Cancer Institute

Charles G. Drake, MD, PhD Johns Hopkins University

Lawrence Fong, MD University of California, San Francisco

Stacey Harrelson, RN Carolina Urologic Research Center

Phillip W. Kantoff, MD Memorial Sloan Kettering Cancer Center

Ravi A. Madan, MD National Cancer Institute, National Institutes of Health

William K. Oh, MD Mount Sinai School of Medicine

David J. Peace, MD University of Illinois

Daniel P. Petrylak, MD Yale Cancer Center

Hank Porterfield Alliance for Prostate Cancer Prevention

Oliver Sartor, MD Tulane University School of Medicine

Neal D. Shore, MD, FACS Carolina Urologic Research Center

Susan F. Slovin, MD, PhD Memorial Sloan Kettering Cancer Center

Mark N. Stein, MD, FACP Rutgers Cancer Institute of New Jersey

Johannes Vieweg, MD, FACS Nova Southeastern University
