# Supplementary material for: The Society for Immunotherapy of Cancer consensus statement on immunotherapy for the treatment of prostate carcinoma
Source: J Immunother Cancer. 2016 Dec 20;4:92. doi: 10.1186/s40425-016-0198-x (PMC5170901; doi:10.1186/s40425-016-0198-x)
Supplement: Additional file 2: — Pre-meeting Survey Questions and Responses. (DOCX 30 kb) [file 40425_2016_198_MOESM2_ESM.docx]

| 1. **What best describes your primary role in prostate cancer (select one):** | | | | | | | | |
| --- | --- | --- | --- | --- | --- | --- | --- | --- |
| **Answer Options** | | | | | | | **Response Percent** | |
| Medical Oncologist | | | | | | | 73.3% | |
| Surgical Oncologist | | | | | | | 6.7% | |
| Radiation Oncologist | | | | | | | 0.0% | |
| Nurse | | | | | | | 6.7% | |
| Patient or Patient Advocate | | | | | | | 6.7% | |
| Other (Translational Research) | | | | | | | 6.7% | |
| 1. **Which of the following is the primary focus of your clinical activity (select one):** | | | | | | | | |
| **Answer Options** | | | | | | | **Response Percent** | |
| Management of localized prostate cancer | | | | | | | 0.0% | |
| Management of recurrent/metastatic prostate cancer | | | | | | | 46.7% | |
| Both of the above | | | | | | | 40.0% | |
| Other/Not Applicable | | | | | | | 13.4% | |
| 1. **With which of the following do you have clinical experience (select all that apply):** | | | | | | | | |
| **Answer Options** | | | | | | | **Response Percent** | |
| Chemotherapy | | | | | | | 93.3% | |
| Immunotherapy | | | | | | | 93.3% | |
| Surgery | | | | | | | 20.0% | |
| Androgen deprivation (hormonal) therapies | | | | | | | 93.3% | |
| Targeted therapies | | | | | | | 100.0% | |
| Clinical Trials | | | | | | | 100.0% | |
| None/Not Applicable | | | | | | | 0.0% | |
| 1. **Which of the following FDA-approved agents have you used or recommended for patients with prostate cancer (select all that apply):** | | | | | | | | |
| **Answer Options** | | | | | | | **Response Percent** | |
| Leuprolide / Degarelix / Goserelin | | | | | | | 100.0% | |
| Bicalutamide / Nilutamide / Flutamide | | | | | | | 100.0% | |
| Docetaxel | | | | | | | 93.3% | |
| Enzalutamide | | | | | | | 93.3% | |
| Sipuleucel-T | | | | | | | 86.7% | |
| Abiraterone | | | | | | | 100.0% | |
| Mitoxantrone | | | | | | | 66.7% | |
| Zoledronic acid / Denosumab | | | | | | | 86.7% | |
| Cabazitaxel | | | | | | | 86.7% | |
| Radium-223 | | | | | | | 93.3% | |
| None/Not applicable | | | | | | | 0.0% | |
| 1. **Is there any (FDA-approved) role for immunotherapy in patients with prostate cancer with non-metastatic, non-castrate disease?** | | | | | | | | |
| **Answer Options** | | | | | | | **Response Percent** | |
| No | | | | | | | 85.7% | |
| Yes | | | | | | | 14.3% | |
| **Open ended responses:** 1) PSA relapse (post RP and post EBRT); 2) There should be. | | | | | | | | |
| 1. **What is the approved indication for the treatment of patients with sipuleucel-T (open ended response)?** | | | | | | | | |
| **Open ended responses:** 1) mCRPC with minimal symptoms; 2) Asymptomatic or minimally symptomatic metastatic castration resistant prostate cancer; 3) Don't know; 4) Minimally symptomatic mCRPC; 5) Asx or minimally sx mCRPC; 6) Castrate-resistant, metastatic, minimally-to-asymptomatic; 7) Met CRPC with limited pain; 8) Asymptomatic or minimally symptomatic metastatic CRPC; 9) Treatment of asymptomatic or minimally symptomatic metastatic castrate resistant prostate cancer; 10) Asymptomatic or minimally symptomatic mCRPC; 11) Asx, minimally sx M1CRPC; 12) Asymptomatic or minimally symptomatic castrate metastatic prostate cancer; 13) mCRPC, either asymptomatic or minimally symptomatic; 14) Metastatic castrate resistant prostate cancer | | | | | | | | |
| 1. **In the era of abiraterone approved for treatment of patients with mCRPC prior to docetaxel, and possible soon approval of enzalutamide in this same disease setting, do you believe there is a role for the use of sipuleucel-T in the management of prostate cancer?** | | | | | | | | |
| **Answer Options** | | | | | | | **Response Percent** | |
| No | | | | | | | 0.0% | |
| Yes, Please describe | | | | | | | 100.0% | |
| **Open ended responses:** 1) Low burden and low pace mCRPC; 2) No different mechanism; 3) N/A; 4) Early in mCRPC and if data supports from future trials perhaps in non-met disease/adjuvant setting; 5) Still may be valuable adjunct to those AR targeted therapies, which by the way are non-curative and overlapping in MOA; 6) Early after the identification of metastatic, castrate-resistant disease, likely before any other therapies, and with low volume (PSA) and slower growth disease; 7) Very limited; 8) There is lots of room in the approved indication for multiple therapies for most patients. I typically start with agents shown to improve survival with fewer side effects; 9) After failure of second-line hormone therapies especially low tumor burden, low PSA CRPC patients; 10) If used, would be used early after initial progression on ADT. Enzalutamide would then be my first choice of additional therapies; 11) Foundational for early detected low volume M1CRPC; 12) Could be moved to earlier clinical state; 13) Probably should be used prior to second gen AA; 14) Ideally sipuleucel-T should be administered as soon as possible after metastasis has been confirmed. | | | | | | | | |
| 1. **Do you use clinical laboratory tests to choose/exclude patients for treatment with sipuleucel-T? If so, which tests?** | | | | | | | | |
| **Answer Options** | | | | | | | **Response Percent** | |
| No | | | | | | | 28.6% | |
| Yes, Please describe | | | | | | | 71.4% | |
| **Open ended responses:** 1) PSADT; 2) Hepatic mets; 3) Don't know; 4) PSA velocity, imaging studies; 5) If PSA is rising VERY RAPIDLY, I would probably not use sip T. Also if ALK PHOS is very elevated and rising fast, I would also probably not use sip T; 6) Rapidly rising PSA (e.g. doubling < 3 months, PSA >> 20 ng/mL), and other bad prognostic indicators (e.g. low hgb, high alk phos); also patients who are not candidates for leukapheresis based on lab parameters (low platelets and/or severe anemia); 7) I typically would not give Sip-T to patients with grade 3 liver enzymes or renal function as they would not have been candidates for the phase 3 study; 8) PSA levels can be helpful in directing clinical choice... with avoidance in high PSA with rapid increments; 9) Patients with abnormal blood parameters (i.e., CBC); 10) CBC | | | | | | | | |
| 1. **Do you use the extent of disease (by radiographic / scintigraphic tests) to determine whether or not to treat patients with sipuleucel-T? If so, what criteria do you use?** | | | | | | | | |
| **Answer Options** | | | | | | **Response Percent** | | |
| No | | | | | | | 35.7% | |
| Yes, please describe | | | | | | | 64.3% | |
| **Open ended responses:** 1) Low volume; 2) Tend to avoid bulky adenopathy; 3) N/A; 4) Rapidly progressing, bulky tumors less likely to benefit; 5) Partly. If a patient has lower volume disease on scan, then I would more likely favor trying sip T. If they have a high burden of mets, it might not be my first choice, depending on other factors including PSADT and symptoms; 6) No absolute criteria, but tend to focus on less extensive disease  liver mets would most likely exclude; 7) Rapid radiographic or biochemical disease progression; 8) No visceral mets. | | | | | | | | |
| 1. **Could patients being considered for sipuleucel-T treatment be concurrently treated with a corticosteroid?** | | | | | | | | |
| **Answer Options** | | | | | | | **Response Percent** | |
| No | | | | | | | 30.8% | |
| Yes, please explain. | | | | | | | 69.2% | |
| **Open ended responses:** 1) No one knows the answer. In vitro tests are not the answer; 2) Don’t like to do it but data suggest no difference in immune parameters; 3) Don't know; 4) Data with Abiraterone and Prednisone showed no decline in CD54 expression (release criteria, APC activation); 5) At least based on the trial of concurrent vs seq Abi/Pred and sip T; 6) Low dose (< 5 mg per day equivalent of prednisone); 7) They can be but prob best if not; 8) Possibly. If the patient was receiving a replacement steroid for adrenal insufficiency this would be acceptable. In addition, there is emerging data suggesting that the ability to manufacture product and to mount immune responses is not compromised by 10mg prednisone daily (along with abi). Furthermore, memory cells are much less susceptible to steroid induced death than naive lymphocytes; 9) Low dose prednisone acceptable; 10) Could but would prefer not to; 11) Needs further discussion. Abi/pred concommitant trial was successful for sip-T manufacture; efficacy data being monitored; that said, need more concrete data w/r timing initiation prednisone post sip T administration; 12) Per SMALL data; 13) Patients do not need to be immunocompromised during the leukapharesis and infusion process. | | | | | | | | |
| 1. **Should patients being considered for sipuleucel-T be weaned off corticosteroid therapies (for example, following treatment with abiraterone) prior to beginning treatment?** | | | | | | | | |
| **Answer Options** | | | | | | | **Response Percent** | |
| No | | | | | | | 50.0% | |
| Yes | | | | | | | 50.0% | |
| Explain: | | | | | | |  | |
| **Open ended responses:** 1) Unless the dose is very low; 2) I would think so; 3) Data with Abiraterone and Prednisone showed no decline in CD54 expression (release criteria, APC activation); 4) For same reason; 5) Probably best - although no data really; 6) Not necessarily. If the patient was receiving a replacement steroid for adrenal insufficiency this would be acceptable. In addition, there is emerging data suggesting that the ability to manufacture product and to mount immune responses is not compromised by 10mg prednisone daily (along with abi). Furthermore, memory cells are much less susceptible to steroid induced death than naive lymphocytes; 7) Not required for low dose prednisone; 8) Yes; 9) IMPACT trial required 30 day wean, but not sure about the scientific validation for that requirement; 10) If possible; 11) See above answer please. | | | | | | | | |
| 1. **What is the preferred (in your opinion) sequence of agents for the management of patients with minimally symptomatic (or asymptomatic) metastatic, castration-resistant prostate cancer? (Number in order):** | | | | | | | | |
| **Answer Options** | **1** | **2** | **3** | **4** | **5** | | | **Rating Average** |
| Abiraterone | 4 | 3 | 6 | 0 | 1 | | | 2.36 |
| Enzalutamide | 0 | 10 | 3 | 1 | 0 | | | 2.36 |
| Sipuleucel-T | 10 | 0 | 3 | 0 | 1 | | | 1.71 |
| Docetaxel | 0 | 1 | 0 | 10 | 3 | | | 4.07 |
| Radium-223 | 0 | 0 | 2 | 3 | 9 | | | 4.50 |
| 1. **Do you ever use sipuleucel-T in combination with another agent (besides a GnRH agonist / antagonist and possibly skeletal related event mitigating agent)? If so, which agent?** | | | | | | | | |
| **Answer Options** | | | | | | | | **Response Percent** |
| No | | | | | | | | 50.0% |
| Yes, Please describe | | | | | | | | 50.0% |
| **Open ended responses:** 1) nilutamide or bicalutamide or enzalutamide; 2) N/A; 3) Enzalutamide; 4) I will consider with concurrent AR targeted therapy, which may include older antiandrogens, but also enza or abi/pred; 5) Enzalutamide in select patients (with more advanced or more agressive disease); 6) Xgeva; 7) In clinical trials, yes. I am unsure of the standard practice in the clinic. | | | | | | | | |
| 1. **With what agents used for treating prostate cancer do you think it is reasonable to combine with sipuleucel-T? (select all that apply):** | | | | | | | | |
| **Answer Options** | | | | | | | | **Response Percent** |
| Surgical or medical castration | | | | | | | | 85.7% |
| Bicalutamide / Nilutamide / Flutamide | | | | | | | | 71.4% |
| Zoledronic acid | | | | | | | | 78.6% |
| Denosumab | | | | | | | | 78.6% |
| Other (please describe): | | | | | | | | 42.9% |
| **Open ended responses:** 1) enzalutamide; 2) enzalutamide; 3) N/A; 4) Enzalutamide, Abiraterone; 4) Enzalutamide; 5) GNRH antagonists, Abi(with or without pred), Enza. | | | | | | | | |
| 1. **Which clinical labs do you obtain in patients undergoing treatment with sipuleucel-T, and at what frequency? (Please type in the frequency ONLY for those labs that you obtain. If your response is “None/not applicable” then write in “none” in that box.)** | | | | | | | | |
| **Answer Options** | | | | | | | | **Response Percent** |
| **CBC** | | | | | | | | **78.6%** |
| **Frequency:** 1) q 2 weeks during Rx; 2) q 3 weeks; 3) Monthly; 4) Monthly; 5) Every two weeks during treatment (for leukapheresis eligibility), then every 1-3 months; 6) Baseline; 7) q3m; 8) Yes; 9) Monthly; 10) q month; 11) Prior to each leukapheresis, | | | | | | | | |
| **Chem 7** | | | | | | | | **78.6%** |
| **Frequency:** 1) None; 2) Monthly; 3) Monthly; 4) Only creatinine, every 1-3 months; 5) Baseline; 6) q3m; 7) q 2 weeks; 8) yes; 9) monthly; 10) q month; 11) Prior to each leukapharesis | | | | | | | | |
| **LDH** | | | | | | | | **71.4%** |
| **Frequency:** 1) None; 2) Monthly; 4) Monthly; 5) q1-3 months; 6) Baseline; 7) None; 8) Yes; 9) Monthly; 10) None; 11) q 2 weeks during Rx | | | | | | | | |
| **PSA** | | | | | | | | **78.6%** |
| **Frequency:** 1) q 2 weeks during Rx; 2) None; 3) Monthly; 4) Monthly; 5) q month; 6) Baseline; 7) q3m; 8) yes; 9) Monthly; 10) q 3 month; 11) None | | | | | | | | |
| **Liver enzymes** | | | | | | | | **64.3%** |
| **Frequency:** 1) None; 2) Monthly; 3) Monthly; 4) None; 5) Baseline; 6) q3m; 7) Yes; 8) Monthly; 9) None | | | | | | | | |
| **Alkaline phosphatase** | | | | | | | | **64.3%** |
| **Frequency:** 1) Monthly; 2) Monthly; 3) q month; 4) Baseline; 5) q3m; 6) Yes; 7) Monthly; 8) None | | | | | | | | |
| **Other** | | | | | | | | **42.9%** |
| 1) None; 2) None; 3) None; 4) None; 5) Calcium levels q 2 weeks; 6) None | | | | | | | | |
| **None/Not applicable** | | | | | | | | **28.6%** |
| 1. **Is there any potential toxicity for which you monitor patients during or after treatment with sipuleucel-T? Please describe:** | | | | | | | | |
| **Answer Options** | | | | | | | | **Response Percent** |
| No | | | | | | | | 28.6% |
| Yes, Please describe | | | | | | | | 71.4% |
| **Open ended responses:** 1) Line infections are the #1 problem; 2) Chills during infusion; 3) N/A; 4) Infusion reactions; 5) Chills; 6) Infusion type rx; 7) Infusion reactions, anaphylaxis; 7) Flu like syndrome; 8) Minimal, cytokine related; 9) Occasionally patients experience rigors- treat with Demerol, monitor temperature, monitor calcium levels, assess central lines for patency, monitor for possible infections. | | | | | | | | |
| 1. **After treatment with sipuleucel-T, at what time point do you obtain the first (open ended response):** | | | | | | | | |
| **Answer Options** | | | | | | | | **Response Percent** |
| **Serum PSA blood test?** | | | | | | | |  |
| **Open ended responses:** 1) monthly; 2) 1 month; 3) N/A; 4) 1 month; 5) 1 month; 6) Monthly; 7) 3 mos; 8) Generally 3 months; 9) 1 m; 10) 1 month; 11) 2-3 months; 12) one month; 13) 3 months; 14) 3 months | | | | | | | | |
| **Radiographic tests (CT scans and/or bone scans)?** | | | | | | | |  |
| **Open ended responses:** 1) Per sxs and PSADT; 2) Depends on PDA; 3) N/A; 4) Based on subsequent treatment; 5) 3-6 months; 6) At the time of new symptoms and at intervals not more than every 6 months; 7) 3-6 months; 8) As clinically indicated (based on PSA and symptoms); 9) 3 m; 10) Varies, 1 to 3 months; 11) 3-4 months; 12) 3-4 months; 13) 3 months; 14) 3-6 months. | | | | | | | | |
| 1. **Is there any role for clinical or other laboratory monitoring of patients after treatment with sipuleucel-T as prognostic information or to determine when to start other therapy?** | | | | | | | | |
| **Answer Options** | | | | | | | | **Response Percent** |
| No | | | | | | | | 35.7% |
| Yes, Please describe | | | | | | | | 64.3% |
| **Open ended responses:** 1) As above; 2) CT scans; 3) N/A; 4) Usu PSA, certainly symptoms and maybe scans; 5) Possibly eosinophils as marker of prognostic information, but no lab tests would definitively indicate time to start other therapy, unless poor change in trajectory (e.g. new rapid change in PSA and other markers might favor beginning AR-targeted agent, but would still get scans. We typically do not start new therapy with lab change only in the absence of new symptoms and/or change in radiographic findings; 6) EOD; 7) I typically follow patients clinically and with PSA. Typically, PSA will continue to rise and may be used in clinical decision making along with symptoms and side effects of other therapies / patient wishes; 8) PSA and radiologic findings useful but interpreted cautiously in light of protracted latency of immunization effects; 9) Antigen-antibody panels and/or gene signatures to predict responders | | | | | | | | |
| 1. **How long do you wait after the last biweekly treatment with sipuleucel-T before considering starting another therapy (open ended response)?** | | | | | | | | |
| **Open ended responses:** 1) I have no wait; 2) Depends on clinical course; 3) N/A; 4) No need to wait. Move on to next Tx immediately; 5) 4 weeks; 6) At least one month; 7) Depends on POD; 8) Variable depending on clinical situation and what modality is to be started next. I often come in with enzalutamide either as early as immediately following or sometime concurrent with Sip-T; 9) As long as possible if patient's symptoms and clinical status don't intercede; 10) 1 to 3 months; 11) 4-8 weeks; 12) 3-4 months; 13) As long as possible; 14) No set time as long as the patient is clinically stable. | | | | | | | | |
| **How do you determine that it is time to start another treatment after having used sipuleucel-T?** | | | | | | | | |
| **Open ended responses:** 1) I start another therapy regardless of progression; 2) Scan changes; 3) N/A; 4) The data suggest there will be no short term change in TTP, thus you should move on to next tx in most cases; 5) PSADT, symptoms, scans; 6) New symptoms related to disease - definitely start; radiographic changes + rise in PSA, probably start; typically do not use PSA only; 7) POD; 8) Based on disease related symptoms worsening (definately add therapy), progression on imaging (strongly recommend adding new therapy), PSA kinetics worsening (recommend adding new therapy) or discussion with patient; 9) Progressive symptoms or clinical deterioration will sometimes dictate early intervention. Relentless progression beyond 6 months (based on radiological or biochemical features) will also lead to intervention; 10) Pace of disease progression; 11) Serologic, radiographic,symptomatic parameters Trial options Approved agents CRPC; 12) Scan progression of rapid PSA doubling time; 13) PSA, radiographic or symptomatic progression; 14) Disease progression or worsening symptoms. | | | | | | | | |
| **Please list other immunotherapy agents, and stage of disease, not FDA-approved, of which you are aware that have demonstrated some activity in phase II/III trials.** | | | | | | | | |
| **Open ended responses:** 1) Ipi Prostvac; 2) Prostavac; 3) N/A; 4) Prostvac, Ipilimumab, GVAX; 5) Ipilimumab, met CRPC; 6) Prostvac - evidence of activity in Phase II - phase III trials pending - in mCRPC Ipilimumab - did not meet phase III endpoint, but close; other trials anticipated - likely mCRPC GVAX - no real phase II trials; closed early in phase III Northwest Biotherapeutics - had phase II trial data in CRPC using PSMA peptide-loaded vaccine; was starting phase III trials, but company never pursued; 7) Anti-PSMA-radiolabeled and/or drug conjugate PSA vaccine; 8) Ipilimumab (mCRPC), Prostvac (mCRPC); 9) Prostvac-VF CRPC; GVAX +ipilimumab CPRC; ipilimumab (chemo naive CPRC) tasquinimod chemo naive CRPC; adenoPSA vaccine mCRPC; 10) Prostvac, Ipilimumab; 11) PROSTVAC Ipilumimab PDL-1 & PD1; 12) ProstVac, CAR+T cells; 13) ProstVac VF, G-vax, Madison vaccines DNA vaccine, Innovio, Ultimovax; 14) Prostvac Phase III trial is indicated for mCRPC | | | | | | | | |
