# Supplementary material for: The Society for Immunotherapy of Cancer consensus statement on immunotherapy for the treatment of prostate carcinoma
Source: J Immunother Cancer. 2016 Dec 20;4:92. doi: 10.1186/s40425-016-0198-x (PMC5170901; doi:10.1186/s40425-016-0198-x)
Supplement: Additional file 3: — Comments Recieved during Review. (DOCX 12 kb) [file 40425_2016_198_MOESM3_ESM.docx]

**Appendix IV: Comments from Open Review**

Comments from the open review of this consensus statement.

| **Comment Date** | **Comment** |
| --- | --- |
| August 7, 2016 | 1. Agree with the conclusion that more results are needed as well as patient related characteristics and predictive immunological parameters of clinical benefit.  2. Agree that combinations must be studied using Sipuleucel-T since on theoretical grounds a dendritic cell vaccine by itself not harboring a high T cell avidity target and not addressing known mechanisms of immune system evasion would have very little chance of benefit.  3. Future combinations should not be limited to early disease but rather to the smaller subset populations that will emerge to benefit from immunotherapy as we have seen with our soon to be published anti-PD1 data.  4. I would argue the benefit of this DC vaccine alone is still questionable despite the statistics, especially given its target population “early” in disease. For a low antigenic tumor like prostate cancer early disease may actually not be the ideal time for immunotherapy (as argued by Hans Schreiber). In fact, there hasn’t been overwhelming results of immunotherapy seen in the adjuvant setting as there has been in the metastatic setting so to argue immunotherapy is better earlier in disease is not without proof. The increase in TILS reported by Larry Fong are of interest but may be deceptive as low avidity cells that cannot control disease without additional help.  5. Our soon to be published anti-PD1 data does show activity in a small population of patients which I believe is soft tissue limited raising the issue of concurrent management of bone metastases which may be more difficult for the immune system to reach. Also responders may have characteristics seen in other cancers regarding situations related to a higher mutation rate and number which will bear out in future studies. |
