# Supplementary material for: The Society for Immunotherapy of Cancer consensus statement on immunotherapy for the treatment of prostate carcinoma
Source: J Immunother Cancer. 2016 Dec 20;4:92. doi: 10.1186/s40425-016-0198-x (PMC5170901; doi:10.1186/s40425-016-0198-x)
Supplement: Additional file 4: — Annotated Bibliography. (PDF 231 kb) [file 40425_2016_198_MOESM4_ESM.pdf]

CANCER IMMUNOTHERAPY GUIDELINES (PROSTATE)

An Annotated Bibliography of the Literature (in order of topic)

With Authors Additions

SOCIETY FOR IMMUNOTHERAPY OF CANCER

JULY 3, 2015

Table of Contents

TOPIC: Sipeuleucel-T 4
TOPIC: Prostate cancer and ipilimumab 5
TOPIC: Prostate cancer and vaccine 5
TOPIC: Prostate cancer and immunotherapy 6
TOPIC: ASCO Guidelines 7
TOPIC: Authors Additions 7

Note: The bibliography is organized in topic order and then in alphabetical order by author. Duplicates and irrelevant entries have been removed in this bibliography and selected references have been added.

The search could be summarized as follows:

- **Searches from 2006 to 2014, conducted on 9/29/14**
- **Search terms: sipuleucel-T, prostate cancer and ipilimumab, prostate cancer and vaccine, prostate cancer and immunotherapy**
- **We then excluded review articles**
- **We included the meta-analyses of large trials, and guidelines reports**
- **We included the clinical trials that were phase II or higher, and excluded phase I or phase I/II trials**

Note: The bibliography is organized in topic order and then in alphabetical order by author. Duplicates and irrelevant entries have been removed in this bibliography and selected references have been added. The original search was conducted on 9/29/14 in the sequence and with the limits as follows:

Prostate Cancer Immunotherapy Literature Searches Conducted September 29, 2014												
**Search Terms**	**Date Limits**	**Limits**	**Search Field Tags**	**Date Search Completed**	**Total Refs Identified**	**File Name with Dupes/File Name without Dupes/File Name without Irrelevants and addons.**	**EndNote record numbers**	**total records found**	**total # dupes**	**Resulting # of records in bibliography**	**Total # Iirrelevant records deleted**	**Resulting # of records in bibliography**
Sipuleucel-T	2006-2014	("sipuleucel-T"[Supplementary Concept] OR "sipuleucel-T"[All Fields] OR "sipuleucel t"[All Fields]) AND ((Clinical Trial[ptyp] OR Meta-Analysis[ptyp] OR Randomized Controlled Trial[ptyp] OR Controlled Clinical Trial[ptyp] OR Practice Guideline[ptyp]) AND ("2006/01/01"[PDAT] : "2014/12/31"[PDAT]) AND "humans"[MeSH Terms])	Supplementary Concept, All Fields, Publication Type, Publication Date, MeSH Terms	9/29/2014	14	Prostate Cancer 092914.enl/Prostate Cancer w Dupes Removed 092914.enl/Prostate cancer 10212014 wDupes&Irrelevents removed and addons.enl	1-14	14	0	14	4	10
Prostate Cancer and ipilimumab	2006-2014	prostatic neoplasms[MeSH Terms] AND "ipilimumab"[Supplementary Concept] AND ((Clinical Trial[ptyp] OR Meta-Analysis[ptyp] OR Randomized Controlled Trial[ptyp] OR Controlled Clinical Trial[ptyp] OR Practice Guideline[ptyp]) AND ("2006/01/01"[PDAT] : "2014/12/31"[PDAT]) AND "humans"[MeSH Terms])	MeSh Terms, Supplementary Concept, Publication Date	9/29/2014	8	Prostate Cancer 092914.enl/Prostate Cancer w Dupes Removed 092914.enl/Prostate cancer 10212014 wDupes&Irrelevents removed and addons.enl	15-22	8	0	8	7	1
Prostate cancer and [vaccine OR therapeutic vaccine]	2006-2014	("prostatic neoplasms"[MeSH Terms] AND "vaccines"[MeSH Terms]) AND ((Clinical Trial[ptyp] OR Meta-Analysis[ptyp] OR Randomized Controlled Trial[ptyp] OR Controlled Clinical Trial[ptyp] OR Practice Guideline[ptyp]) AND ("2006/01/01"[PDAT] : "2014/12/31"[PDAT]) AND "humans"[MeSH Terms])	MeSH Terms, Publication Type, Publication Date	9/29/2014	69	Prostate Cancer 092914.enl/Prostate Cancer w Dupes Removed 092914.enl/Prostate cancer 10212014 wDupes&Irrelevents removed and addons.enl	23-91	69	11	58	45	13
Prostate cancer and immunotherapy	2006-2014	prostatic neoplasms[MeSH Terms] AND "immunotherapy"[MeSH Terms] AND ((Clinical Trial[ptyp] OR Meta-Analysis[ptyp] OR Randomized Controlled Trial[ptyp] OR Controlled Clinical Trial[ptyp] OR Practice Guideline[ptyp]) AND ("2006/01/01"[PDAT] : "2014/12/31"[PDAT]) AND "humans"[MeSH Terms])	MeSH Terms, Publication Type, Publication Date	9/29/2014	53	Prostate Cancer 092914.enl/Prostate Cancer w Dupes Removed 092914.enl/Prostate cancer 10212014 wDupes&Irrelevents removed and addons.enl	92-144	53	43	10	7	3
ASCO Guideline added by Taskforce	N/A	Per Dr. Gulley email to Sheryl Konrad 10/21/14 8:27 am: Sheryl, We have reviewed the references provided and decided to stick with phase II or above studies. The highlighted articles here represent the references we want to keep for this project. In addition, there is one more, an ASCO guideline that came out last month: http://www.ncbi.nlm.nih.gov/pubmed/?term=basch+virgo Please let us know if you have any questions. James	N/A	10/21/2014	1	Prostate Cancer 092914.enl/Prostate Cancer w Dupes Removed 092914.enl/Prostate cancer 10212014 wDupes&Irrelevents removed and addons.enl	145	1	0	1	0	1
		(GVAX and PROSTVAC should be picked up by prostate cancer and vaccine, but these, like Ipilimumab, will just be for potential future agents anyway although there may be supportive data here for some concepts similar with immunotherapy / therapeutic vaccines										
Authors Additions	N/A	Sheryl Konrad email dated 5/11/15. Queries resolved 6/22/15.	N/A	6/21/15 and 7/3/15	10	Prostate Cancer 07032015.enl	146-155	10	4	6		6
						TOTALS		155	58	97	63	34

NOTE: IN THE BIBLIOGRAPHY, THE NUMBER IN BRACKETS IS THE RECORD NUMBER IN ENDNOTE (e.g., 10 is the record number for the first item in this bibliography). This is the correct number to use for identifying references in the manuscript during the manuscript draft stages.

TOPIC: Sipeuleucel-T

1 [10].Beer, T. M., G. T. Bernstein, et al. (2011). "Randomized trial of autologous cellular immunotherapy with sipuleucel-T in androgen-dependent prostate cancer." Clin Cancer Res **17**(13): 4558-4567.

2 [1]. Beer, T. M., P. F. Schellhammer, et al. (2013). "Quality of life after sipuleucel-T therapy: results from a randomized, double-blind study in patients with androgen-dependent prostate cancer." Urology **82**(2): 410-415.

3 [5]. Botrel, T. E., O. Clark, et al. (2012). "Immunotherapy with Sipuleucel-T (APC8015) in patients with metastatic castration-refractory prostate cancer (mCRPC): a systematic review and meta-analysis." Int Braz J Urol **38**(6): 717-727.

4 [6]. Flanigan, R. C., A. J. Polcari, et al. (2013). "An analysis of leukapheresis and central venous catheter use in the randomized, placebo controlled, phase 3 IMPACT trial of Sipuleucel-T for metastatic castrate resistant prostate cancer." J Urol **189**(2): 521-526.

5 [12].Higano, C. S., P. F. Schellhammer, et al. (2009). "Integrated data from 2 randomized, double-blind, placebo-controlled, phase 3 trials of active cellular immunotherapy with sipuleucel-T in advanced prostate cancer." Cancer **115**(16): 3670-3679.

6 [11].Kantoff, P. W., C. S. Higano, et al. (2010). "Sipuleucel-T immunotherapy for castration-resistant prostate cancer." N Engl J Med **363**(5): 411-422.

7 [7]. Mohler, J. L., A. J. Armstrong, et al. (2012). "Prostate cancer, Version 3.2012: featured updates to the NCCN guidelines." J Natl Compr Canc Netw **10**(9): 1081-1087.

8 [2]. Schellhammer, P. F., G. Chodak, et al. (2013). "Lower baseline prostate-specific antigen is associated with a greater overall survival benefit from sipuleucel-T in the Immunotherapy for Prostate Adenocarcinoma Treatment (IMPACT) trial." Urology **81**(6): 1297-1302.

9 [8]. Sheikh, N. A., D. Petrylak, et al. (2013). "Sipuleucel-T immune parameters correlate with survival: an analysis of the randomized phase 3 clinical trials in men

with castration-resistant prostate cancer." Cancer Immunol Immunother **62**(1): 137-147.

10 [13]. Small, E. J., P. F. Schellhammer, et al. (2006). "Placebo-controlled phase III trial of immunologic therapy with sipuleucel-T (APC8015) in patients with metastatic, asymptomatic hormone refractory prostate cancer." J Clin Oncol **24**(19): 3089-3094.

TOPIC: Prostate cancer and ipilimumab

1 [15]. Kwon, E. D., C. G. Drake, et al. (2014). "Ipilimumab versus placebo after radiotherapy in patients with metastatic castration-resistant prostate cancer that had progressed after docetaxel chemotherapy (CA184-043): a multicentre, randomised, double-blind, phase 3 trial." Lancet Oncol **15**(7): 700-712.

TOPIC: Prostate cancer and vaccine

1 [76]. Acres, B. (2007). "Cancer immunotherapy: phase II clinical studies with TG4010 (MVA-MUC1-IL2)." J BUON **12 Suppl 1**: S71-75.

2 [75]. Amato, R. J., N. Drury, et al. (2008). "Vaccination of prostate cancer patients with modified vaccinia ankara delivering the tumor antigen 5T4 (TroVax): a phase 2 trial." J Immunother **31**(6): 577-585.

3 [89]. Arlen, P. M., J. L. Gulley, et al. (2006). "A randomized phase II study of concurrent docetaxel plus vaccine versus vaccine alone in metastatic androgen-independent prostate cancer." Clin Cancer Res **12**(4): 1260-1269.

4 [48]. Draube, A., N. Klein-Gonzalez, et al. (2011). "Dendritic cell based tumor vaccination in prostate and renal cell cancer: a systematic review and meta-analysis." PLoS One **6**(4): e18801.

5 [70]. Dreicer, R., W. M. Stadler, et al. (2009). "MVA-MUC1-IL2 vaccine immunotherapy (TG4010) improves PSA doubling time in patients with prostate cancer with biochemical failure." Invest New Drugs **27**(4): 379-386.

6 [62]. Gulley, J. L., P. M. Arlen, et al. (2010). "Immunologic and prognostic factors associated with overall survival employing a poxviral-based PSA vaccine in metastatic castrate-resistant prostate cancer." Cancer Immunol Immunother **59**(5): 663-674.

7 [30]. Harrop, R., F. Chu, et al. (2013). "Vaccination of castration-resistant prostate

cancer patients with TroVax (MVA-5T4) in combination with docetaxel: a randomized phase II trial." Cancer Immunol Immunother **62**(9): 1511-1520.

8 [60]. Kantoff, P. W., T. J. Schuetz, et al. (2010). "Overall survival analysis of a phase II randomized controlled trial of a Poxviral-based PSA-targeted immunotherapy in metastatic castration-resistant prostate cancer." J Clin Oncol **28**(7): 1099-1105.

9 [74]. Madan, R. A., J. L. Gulley, et al. (2008). "Analysis of overall survival in patients with nonmetastatic castration-resistant prostate cancer treated with vaccine, nilutamide, and combination therapy." Clin Cancer Res **14**(14): 4526-4531.

10 [59]. Noguchi, M., T. Kakuma, et al. (2010). "A randomized phase II trial of personalized peptide vaccine plus low dose estramustine phosphate (EMP) versus standard dose EMP in patients with castration resistant prostate cancer." Cancer Immunol Immunother **59**(7): 1001-1009.

11 [80]. Noguchi, M., T. Mine, et al. (2007). "Combination therapy of personalized peptide vaccination and low-dose estramustine phosphate for metastatic hormone refractory prostate cancer patients: an analysis of prognostic factors in the treatment." Oncol Res **16**(7): 341-349.

12 [45]. Noguchi, M., F. Moriya, et al. (2012). "Phase II study of personalized peptide vaccination for castration-resistant prostate cancer patients who failed in docetaxel-based chemotherapy." Prostate **72**(8): 834-845.

13 [26]. Noguchi, M., F. Moriya, et al. (2013). "A phase II trial of personalized peptide vaccination in castration-resistant prostate cancer patients: prolongation of prostate-specific antigen doubling time." BMC Cancer **13**: 613.

TOPIC: Prostate cancer and immunotherapy

1 [98]. Cookson, M. S., B. J. Roth, et al. (2013). "Castration-resistant prostate cancer: AUA Guideline." J Urol **190**(2): 429-438.

2 [132]. Slovin, S. F., G. Ragupathi, et al. (2007). "A polyvalent vaccine for high-risk prostate patients: "are more antigens better?"." Cancer Immunol Immunother **56**(12): 1921-1930.

3 [133]. Small, E. J., N. Sacks, et al. (2007). "Granulocyte macrophage colony-stimulating factor--secreting allogeneic cellular immunotherapy for hormone-refractory prostate cancer." Clin Cancer Res **13**(13): 3883-3891.

TOPIC: ASCO Guidelines

1 [145]. Basch, E., D. A. Loblaw, et al. (2014). "Systemic therapy in men with metastatic castration-resistant prostate cancer: american society of clinical oncology and cancer care ontario clinical practice guideline." J Clin Oncol **32**(30): 3436-3448.

TOPIC: Authors Additions

1 [148]. Cookson, M. S., W. T. Lowrance, et al. (2015). "Castration-resistant prostate cancer: AUA guideline amendment." J Urol **193**(2): 491-499.

2 [147]. Gandhi, N. M., A. Morales, et al. (2013). "Bacillus Calmette-Guerin immunotherapy for genitourinary cancer." BJU Int **112**(3): 288-297.

3 [146]. Guinan, P. D., T. John, et al. (1982). "Adjuvant immunotherapy (BCG) in stage D prostate cancer." Am J Clin Oncol **5**(1): 65-68.

4 [154]. Podrazil, M., D. Rozkova, et al. (2014). "Combined chemoimmunotherapy of castrate-resistant prostate cancer with dendritic-cell based vaccine DCVAC/Pca." ASCO Meeting Abstracts **32**(15_suppl): 3095.

5 [155]. Small, E. J., C. S. Higano, et al. (2013). "Relationship of sipuleucel-T with time to first use of opioid analgesics (TFOA) in patients (pts) with asymptomatic or minimally symptomatic metastatic castration-resistant prostate cancer (mCRPC) on the IMPACT trial." ASCO Meeting Abstracts **31**(6_suppl): 74.

6 [153]. Spisek, R., M. Podrazil, et al. (2014). "Cancer immunotherapy of patients with the biochemical relapse of the prostate cancer using dendritic cell-based vaccine DCVAC/PCa." ASCO Meeting Abstracts **32**(15_suppl): 3099.
